# Treatment of Chancroid by the Potassate of Sozoiodol

**Published:** 1890-03

**Authors:** Charles Szadek

**Affiliations:** Kieff, Russia


### THE TREATMENT OF CHANCROID BY THE POTASSATE OF SOZOIODOL.

BY CHARLES SZADEK, M. D., KIEFF, RUSSIA.

The application of iodoform is a favorite method of treatment of chancres with many, but there are certain disadvantages attached to it which sometimes render its use impossible. There is in the first place the disagreeable odor which often shuts off the patients under treatment from the society of their fellowmen. Secondly, the drug is not well borne by every one and it occasionally excites an erythema or an eczematous eruption which compels its withdrawal; and thirdly, in ulcers with overhanging edges, the use of iodoform sometimes excites such exuberant granulations that they spring up and press against the undermined edges of the ulcer, preventing the escape of pus formed beneath.

I have recently called attention to the beneficial action of the potassate of Sozoiodol in the treatment of chancroids. This medicament has been used by *Fritsch*, *Seifert* and *Lassar* as a substitute for iodoform in the treatment of the various affections of the nose, larynx and skin, with results, confirming the good opinion held of the drug by *Langaardt* (Therap. Monatshefte, 1888, September). Recently *Kopytowski* of Warshaw, Poland, reports nine cases of soft chancre for which the salts of Sozoiodol were used with the happiest results.

$$\text{Sozoiodol } C_6\,H_2 \begin{cases} I_2 \\ H\,O \\ S\,O_3H \end{cases}$$

is a new product of chemical synthesis and contains 52 per cent.

of iodine, 20 per cent. of carbolic acid and 7 per cent. of sulphur. Its potassium salt is in the form of finest crystalline scales and dissolves to the extent of 2 per cent. in water, the sodium to 7 or 8 per cent.; the zinc salt dissolves easily, the mercury compound with difficulty; they are odorless; chemical and therapeutic researches prove its exact identity with iodoform.

In my private practice during last year I have used the potassate of Sozoiodol in substance or with creolin (1%), Bism. subnitricum (5–10%) in 42 cases of chancroid with prompt success. The following are the usual prescribed formulæ:

*Rp:* Sozoiodolkali.... 4, 0.

Ds. Pulvis.

*Rp:* Sozoiodolkali.... 10, 0.

Creolini........ 0, 1.

Mf. Pulvis.

*Rp:* Sozoiodolkali - - - 10, 0.

Bism. Subnitrici - - 0, 5—1, 0.

Mf. Pulvis.

The powdered substance is applied to the ulcer and covered with a thin layer of wadding, over which is placed the ordinary dressing; the dressing is changed every twety-four hours; in the case of soft chancres which are so situated that the dressing becomes saturated with urine, or which secretes profusely, it may be necessary to renew the drug twice daily. In the great majority of cases in five to seven days the ulcer loses entirely its specific characteristic; when this result has been obtained, that is, when the lesion has been converted into a simple ulcer, the applications of potassate of Sozoiodol should be suspended, and boracic-acid employed. Under this treatment, the ulcer becomes covered with healthy granulations, and cicatrization proceeds with great rapidity.

The merits of the new substitute for iodoform are that it is odorless, or nearly so, tasteless, produces no constitutional effects; it is antiseptic, a promoter of granulation and healing, and it arrests suppuration and deodorizes foul secretions. As a substitute for iodoform in the treatment of chancroid, the new drug has positively the same therapeutic effect as iodoform.

*Kieff, Russia, Theater St. 2, Dec. 18th, 1889.*

